# Ultrasound-guided acupotomy: a paradigm shift toward precision and safety in carpal tunnel syndrome therapy

**DOI:** 10.3389/fmed.2026.1785342

**Published:** 2026-03-25

**Authors:** Hai-kun Wang, Jian-cheng Zhang, Guo-hui Wu, Da-quan Yan, Feng-tao Zhou

**Affiliations:** 1 Department of Orthopedics, Shenzhen Pingle Orthopedic Hospital (Shenzhen Pingshan Traditional Chinese Medicine Hospital), Shenzhen, China; 2 Department of Ultrasound, Shenzhen Pingle Orthopedic Hospital (Shenzhen Pingshan Traditional Chinese Medicine Hospital), Shenzhen, China

**Keywords:** carpal tunnel syndrome, image-guided intervention, minimally invasive therapy, patient safety, transverse carpal ligament release, ultrasound-guided acupotomy

## Abstract

Effective management of carpal tunnel syndrome requires balancing therapeutic efficacy with procedural safety. Traditional acupotomy, while minimally invasive, is limited by its dependence on non-visualized techniques that can lead to inaccurate anatomical targeting and potential complications. Ultrasound guidance overcomes these limitations by enabling real-time visualization of key anatomical structures during the procedure. This approach facilitates precise, targeted release of the transverse carpal ligament while minimizing risks to neurovascular tissues. This perspective outlines the theoretical basis, clinical evidence, and practical implementation of ultrasound-guided acupotomy. Available data suggest this method enhances functional recovery while improving patient experience through greater procedural transparency. The integration of imaging guidance provides a pathway toward standardizing this minimally invasive intervention. Further validation through rigorous clinical studies and the establishment of structured training protocols will support its appropriate integration into clinical practice for carpal tunnel syndrome management.

## 1 Introduction

Carpal tunnel syndrome (CTS) is the most prevalent compressive peripheral neuropathy, imposing a substantial socioeconomic burden (1, 2). The condition results from median nerve compression within the carpal tunnel, leading to sensory disturbances, pain, numbness, and eventual motor deficits in the hand (1). These symptoms significantly impair daily function and occupational capacity. Its incidence continues to rise, influenced by an aging population and widespread repetitive hand use, establishing CTS as a major public health concern (3). Current management follows a stepwise protocol, beginning with conservative measures such as splinting, pharmacotherapy, or steroid injections, and progressing to surgical release in refractory cases (4). However, this conventional approach presents clear limitations. Conservative therapies often provide only temporary or partial relief for moderate-to-severe cases, with high rates of symptom recurrence (4). Surgical intervention, while effective, carries inherent risks of infection, nerve injury, scar formation, and prolonged postoperative recovery, which may deter patient acceptance (5).

Acupotomy, a minimally invasive technique derived from integrated medical principles, has gained traction in managing chronic musculoskeletal pain disorders, including CTS (6). It employs a specialized needle designed to perform simultaneous micro-cutting and soft tissue release, targeting the transverse carpal ligament to decompress the median nerve (7). Compared to conventional surgery, acupotomy offers reduced trauma and faster procedural turnover. Nevertheless, its traditional practice relies heavily on palpation and anatomical landmarks for guidance, constituting an essentially “blind” intervention (8). This non-visualized approach introduces considerable technical uncertainty, particularly given normal anatomical variations and pathological alterations in tissue planes (8). The inability to reliably localize the needle tip relative to critical neurovascular structures elevates the risk of iatrogenic injury and may lead to inconsistent or incomplete ligament release (9). These concerns regarding safety, reproducibility, and efficacy have fueled ongoing debate within the academic community, limiting its integration into standardized treatment pathways.

In the present study, the term “acupotomy” specifically refers to a needle-knife–based percutaneous soft tissue release technique grounded in acupuncture principles (10, 11). This definition distinguishes it from the broader concept of “percutaneous carpal tunnel release,” which encompasses multiple minimally invasive methods designed to divide the transverse carpal ligament (12). Throughout this article, the term “acupotomy” is used consistently to denote the specific needle-knife instrument and technique applied for ligament release, thereby avoiding conceptual overlap between related interventional approaches.

**FIGURE 1:**
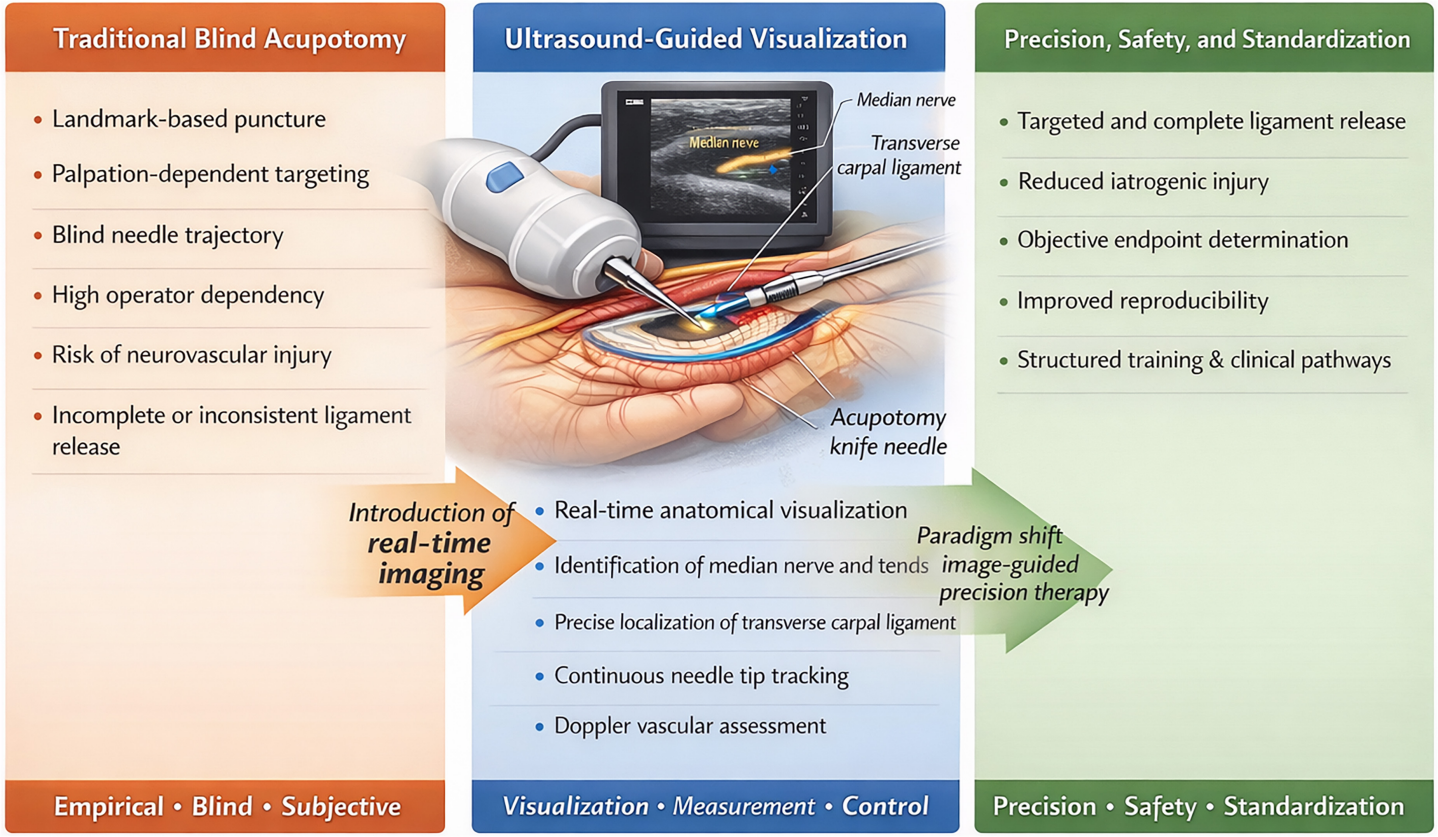
Paradigm shift from blind acupotomy to ultrasound-guided precisious intervention in carpal tunnel syndrome.

The integration of real-time ultrasound imaging represents a potential paradigm shift, addressing the fundamental limitations of traditional acupotomy. Ultrasound guidance transforms the procedure from a blind maneuver into a visually monitored, precise intervention (13) (Figure 1). It enables dynamic visualization of anatomical structures-including the median nerve, flexor tendons, and vasculature-as well as real-time tracking of the needle tip throughout its trajectory (14). This direct visualization significantly redefines the safety and accuracy parameters of the technique: it minimizes the risk of collateral damage by allowing active avoidance of critical structures and ensures targeted delivery of the therapeutic release to the intended site (14). Consequently, ultrasound guidance not only mitigates the primary safety concerns associated with conventional acupotomy but also elevates its potential as a standardized, objective, and reproducible precision therapy (15).

In this perspective, statements derived from empirical methodologies—such as randomized trials, systematic reviews, or comparative studies—are interpreted as evidence-supported observations. In contrast, mechanistic interpretations and implementation proposals are presented as conceptual inferences intended to guide future inquiry, rather than as definitive conclusions. This epistemological framework is essential for contextualizing the subsequent discussion, wherein clinical evidence and technical rationales are integrated to inform evidence-based practice while acknowledging the evolving nature of this therapeutic approach.

This perspective article examines the rationale, technical execution, clinical evidence, and comparative advantages of ultrasound-guided acupotomy for CTS. Furthermore, it discusses future directions for research and clinical implementation, aiming to inform evidence-based practice and foster scholarly discourse in this evolving field.

## 2 Theoretical foundation of the paradigm shift: from empirical anatomy to visualized real-time anatomy

### 2.1 Anatomical complexity of carpal tunnel and imperative for visualization

The carpal tunnel is a confined fibro-osseous canal containing the median nerve, nine flexor tendons, and their synovial sheaths, alongside potential anatomical variants such as a persistent median artery. The spatial relationships among these structures are dynamic and exhibit significant individual variation, which cannot be reliably appreciated through surface landmarks alone (16). High-frequency ultrasonography directly addresses this limitation. It provides real-time, high-resolution visualization of key structures: the thickness and integrity of the transverse carpal ligament, the morphology and echotexture of the median nerve (including sites of compression), tendon motion, and adjacent vasculature (17). This capability transforms static anatomical knowledge into a dynamic, patient-specific navigational map, enabling precise preoperative assessment and intraoperative avoidance of critical structures (18).

### 2.2 Reinterpreted mechanism of ultrasound-guided acupotomy

Ultrasound guidance redefines the therapeutic mechanism of acupotomy from a blind mechanical release to a precise, image-guided intervention. First, it ensures anatomical accuracy (8). The operator can visualize the needle tip in real time, directing it to a specific target layer of the transverse carpal ligament to perform controlled cutting or separation (19). This maximizes therapeutic effect on the ligament while minimizing risk to the underlying median nerve and vessels. Second, it enables continuous procedural control (19). The operator can monitor the needle’s trajectory, depth, and the extent of tissue release, allowing for immediate adjustments. This shifts the procedure from an experience-dependent “estimation” to an image-based “measurement,” ensuring consistency and completeness of the intervention (14, 20).

### 2.3 Philosophical shift: from “blind cutting” to “image-guided precision”

This integration represents a fundamental philosophical transition in clinical approach. Traditional acupotomy is largely anchored in the empirical principle of “treating the tender point,” relying on palpation and practitioner experience. Ultrasound guidance establishes a paradigm of objective, structure-specific targeting (8). The treatment focus moves from a subjective pain point to a directly visualized pathological entity, such as a thickened ligament or a compressed nerve (21). This shift enhances not only safety and efficacy but also the reproducibility and scientific validity of the procedure (22). By providing an objective record of each step, ultrasound guidance facilitates standardized training, rigorous outcome measurement, and multicenter research, thereby advancing acupotomy from an empirical technique to a verifiable, modern interventional discipline (23).

## 3 Technical procedure and considerations for standardization

### 3.1 Selection of ultrasound equipment and needle tools

The successful execution of ultrasound-guided acupotomy depends primarily on appropriate imaging technology and specialized instruments. A high-frequency linear ultrasound transducer (typically ≥ 12 MHz) is essential, as it provides high-resolution visualization of superficial wrist anatomy, clearly depicting skin, subcutaneous tissue, the transverse carpal ligament, and deeper structures including the median nerve and flexor tendons (24). Optimizing ultrasound settings—such as depth, focus, and gain—is necessary to enhance target visualization and minimize needle artifact (25). For needle selection, conventional smooth-surfaced acupotomy needles are often poorly visualized under ultrasound (25). Modified needles designed for enhanced sonographic visibility, such as those with echogenic coatings or specially treated tips, are therefore strongly recommended (26). These modifications allow clear, real-time tracking of the needle tip throughout the procedure, forming the technical basis for precision and safety.

### 3.2 Standardized operational steps

A standardized protocol is essential for ensuring consistent procedural execution and reliable outcomes. First, the patient is placed supine with the affected hand in a supinated position. A systematic ultrasound scan in both transverse and longitudinal planes is performed to identify the transverse carpal ligament, median nerve, and adjacent vascular structures, with key anatomical landmarks marked on the skin (27). Second, an in-plane needle approach is employed to allow continuous visualization of the entire needle shaft and tip (28). The insertion point is chosen at the ulnar or radial border of the ligament, within a safe zone clear of neurovascular structures. Under real-time ultrasound guidance, the needle is advanced to the superficial or thickest region of the transverse carpal ligament (14). Third, a controlled ligament release is performed using techniques such as linear cutting or fan-shaped separation (29). Throughout this step, the transducer is stabilized to maintain clear needle visualization, ensuring precise application of force to the ligament while continuously monitoring the median nerve.

To ensure procedural reproducibility in clinical implementation and facilitate accurate replication in both research and practice, several minimum reporting elements should be explicitly specified (30). These include detailed documentation of the local anesthesia protocol (typically ultrasound-guided periligamentous infiltration), sterile field preparation, and a clear definition of the in-plane approach trajectory (31). Additional technical specifications—such as needle dimensions, blade characteristics, and the number of treatment sessions—should also be systematically reported (32). Furthermore, standardized post-procedural care, encompassing compression protocols, activity modifications, and short-term follow-up assessment, should be clearly described to enable consistent outcome evaluation across different clinical settings and studies (33). Adherence to these standardized operational steps and comprehensive reporting elements enhances procedural safety, reproducibility, and the validity of comparative assessments in future investigations (34).

### 3.3 Intraoperative assessment and endpoint determination

Real-time ultrasound imaging is fundamental for verifying both therapeutic effect and procedural safety. The anatomical endpoint is confirmed by the direct visualization of a definitive separation or discontinuity within the transverse carpal ligament (35). Concurrently, the longitudinal view may show reduced compression on the median nerve, characterized by diminished notching and improved gliding motion (36). A Doppler examination should then be performed to rule out active hemorrhage or vascular compromise in the treated area (37). Finally, a comprehensive scan assesses the integrity of adjacent neurovascular and tendinous structures while reviewing the needle path for any complications (38). This systematic, image-guided evaluation protocol provides an objective basis for concluding the intervention, thereby reducing the likelihood of incomplete or excessive treatment. Furthermore, it creates a standardized, reproducible assessment method crucial for skill training, proficiency verification, and future research.

However, comprehensive endpoint confirmation should integrate both structural and functional ultrasound indicators beyond simple ligament visualization (39). These include measurable separation of the ligament, quantifiable reduction in median nerve cross-sectional area reflecting decompression, and objective restoration of nerve mobility during dynamic assessment (e.g., tendon glide or wrist flexion-extension maneuvers) (39–41). In cases where complete ligament discontinuity is not achieved or where symptoms persist despite apparent anatomical release, staged reassessment or supplementary release procedures may be considered, guided by repeat imaging evaluation to optimize clinical outcomes and minimize the risk of incomplete treatment (39, 40).

## 4 Analysis of clinical evidence and safety profile

### 4.1 Evidence identification and appraisal approach

Given the structured nature of this perspective, the clinical evidence presented was derived from a structured narrative search rather than a systematic review. Literature was retrieved from PubMed and Web of Science, covering publications from January 2000 to December 2025. The search focused on studies addressing ultrasound-guided carpal tunnel interventions, acupotomy or needle-knife release, procedural safety, and functional outcomes. Priority was given to randomized controlled trials, systematic reviews, cadaveric validation studies, and comparative clinical investigations. To support balanced interpretation and minimize selection bias, studies were qualitatively assessed for relevance, methodological rigor, and consistency of reported outcomes.

### 4.2 Review of current clinical studies

Accumulating clinical evidence supports the efficacy and safety of ultrasound-guided acupotomy for CTS (Table 1). Randomized controlled trials indicate that this method leads to better functional outcomes than corticosteroid injection alone, which is attributed to the precise, real-time visualization enabling complete release of the transverse carpal ligament (7, 8). Cadaveric studies further validate the technical feasibility of achieving adequate ligament dissection under ultrasound guidance (8). In clinical comparisons, ultrasound-guided percutaneous release has shown outcomes similar to mini-open surgery, with the advantage of reduced postoperative recovery burden (42). When compared to traditional blind acupotomy, the ultrasound-guided technique results in more consistent improvement in electrophysiological parameters, such as median nerve conduction velocity (43). Recent investigations also suggest that combining this approach with biologic agents may enhance therapeutic outcomes (13). Together, these studies establish ultrasound-guided acupotomy as a precise, effective, and minimally invasive option in the management of CTS.

**TABLE 1:** Comparison of blind acupotomy and ultrasound-guided acupotomy in carpal tunnel syndrome.

Domain	Blind acupotomy	Ultrasound-guided acupotomy
Anatomical targeting	Landmark-based	Real-time visualization
Needle control	Estimated depth	Continuous needle tracking
Safety	Variable, nerve risk	Reduced iatrogenic injury
Release completeness	Operator-dependent	Image-confirmed endpoint
Reproducibility	Low	High
Training feasibility	Experience-based	Standardizable curriculum

### 4.3 Fundamental advancement in safety

Ultrasound guidance substantially improves the safety of acupotomy. Studies confirm that real-time visualization minimizes iatrogenic injury to the median nerve, ulnar artery, and flexor tendons (8). Enhanced visualization also reduces postoperative hematoma incidence by enabling operators to avoid vascular structures (44). The precision afforded by ultrasound further decreases tissue trauma and associated infection risks (45).

Beyond immediate procedural safety, available outcome data demonstrate favorable clinical effectiveness. Reported studies generally show improvements in symptom severity and functional scores over follow-up periods ranging from 3 to 12 months, accompanied by measurable gains in median nerve conduction velocity (46, 47). These functional improvements complement the safety advantages by confirming that enhanced precision translates into meaningful clinical benefits (46). Available data also indicate low complication rates—typically between 1% and 3% for ultrasound-guided procedures—although variability in study outcomes across different designs warrants cautious interpretation when generalizing these findings (46).

Collectively, this evidence supports the position of ultrasound-guided acupotomy as a standardized, minimally invasive procedure with a safety profile and clinical outcomes comparable to other established interventions for carpal tunnel syndrome.

### 4.4 Cost-effectiveness and patient acceptance

From a health-economic standpoint, ultrasound-guided acupotomy is predominantly performed in outpatient settings, lowering direct costs by avoiding operating room use (48). Accelerated functional recovery also reduces indirect socioeconomic burdens by shortening time away from work (48). These economic considerations are primarily applicable to healthcare systems in which office-based ultrasound interventions are operationally feasible and reimbursement frameworks support outpatient minimally invasive procedures (49). Accordingly, the magnitude of potential cost advantages may vary across regions depending on healthcare infrastructure, resource availability, and payment policy structures (49). For patients, preoperative ultrasound visualization enhances anatomical understanding, while intraoperative monitoring increases confidence in procedural safety (50). These factors improve treatment adherence and satisfaction (48, 50). Collectively, ultrasound guidance adds value not only in clinical outcomes but also in psychological, experiential, and economic terms, supporting its broader integration into practice.

## 5 Future directions and multidisciplinary integration

### 5.1 Technology integration and innovation

The evolution of ultrasound-guided acupotomy will benefit from integration with advanced imaging and intelligent systems. Artificial intelligence, particularly deep learning for medical image analysis, may allow automated segmentation of carpal tunnel structures, assist in preoperative planning, provide real-time navigation during needle insertion, and objectively evaluate ligament release (51, 52). These advances could enhance procedural consistency and support clinical decisions. Improvements in ultrasound technology, such as three-dimensional imaging for volumetric visualization and elastography for quantitative tissue stiffness assessment, will further refine anatomical understanding and provide functional biomarkers to guide treatment endpoints (16, 53, 54).

### 5.2 Transformation of education and training

Effective dissemination of this technique requires updated training frameworks. A structured curriculum in ultrasound-guided intervention should be established as a core competency, incorporating high-fidelity simulation for skills practice in probe handling, image interpretation, and needle navigation (55, 56). A competency-based approach ensures proficiency before clinical practice, shortening the learning curve while maintaining safety and quality (55).

### 5.3 Development of a multidisciplinary collaborative framework

Ultrasound-guided acupotomy is inherently multidisciplinary. An integrated clinical model should combine the expertise of sonographers for imaging guidance, pain or rehabilitation specialists for procedural management, and hand surgeons for complex cases (56). Collaboration should also focus on developing standardized clinical pathways based on objective imaging (57). Through robust evidence generated from such teamwork, this technique can be advocated for inclusion in formal treatment guidelines, promoting its adoption as a validated, minimally invasive option in carpal tunnel syndrome management (15, 58).

## 6 Summary

This perspective article delineates the paradigm shift in CTS therapy through ultrasound-guided acupotomy. By integrating real-time dynamic imaging, the technique elevates traditional landmark-dependent acupotomy into a precise, anatomy-based procedure. This enhances the accuracy of transverse carpal ligament release while substantially mitigating risks to the median nerve and adjacent vessels. Thus, it establishes a novel therapeutic option for CTS that is effective, minimally invasive, and safe.

To advance clinical adoption, future efforts should prioritize well-designed randomized controlled trials to generate robust evidence on efficacy and safety. Concurrently, standardized training and procedural guidelines are essential to ensure consistent and appropriate application.

Beyond technical refinement, ultrasound-guided acupotomy represents an evolution in integrated minimally invasive therapy—toward objectivity, standardization, and global relevance. This model also offers a transferrable framework for managing other peripheral nerve entrapment syndromes, such as cubital and tarsal tunnel syndromes.

## Data Availability

The original contributions presented in this study are included in the article/supplementary material, further inquiries can be directed to the corresponding author.
